# MiR-199a-modified exosomes from adipose tissue-derived mesenchymal stem cells improve hepatocellular carcinoma chemosensitivity through mTOR pathway

**DOI:** 10.1186/s13046-019-1512-5

**Published:** 2020-01-02

**Authors:** Guohua Lou, Liang Chen, Caixia Xia, Weina Wang, Jinjin Qi, Aichun Li, Liying Zhao, Zhi Chen, Min Zheng, Yanning Liu

**Affiliations:** 1 State Key Laboratory for Diagnosis and Treatment of Infectious Diseases, National Clinical Research Center for Infectious Diseases, Collaborative Innovation Center for Diagnosis and Treatment of Infectious Diseases, The First Affiliated Hospital, College of Medicine, Zhejiang University, 79# Qingchun Road, 6A-17, Hangzhou, 310003 China; 2 Thyroid Disease Diagnosis and Treatment Center, the First Affiliated Hospital, School of Medicine, Zhejiang University, Hangzhou, China; 3 Department of Infectious Diseases, Affiliated Hangzhou First People’s Hospital, Zhejiang University School of Medicine, Hangzhou, China; 4 Department of Infectious Disease, the Second Affiliated Hospital, Zhejiang University School of Medicine, Hangzhou, China

**Keywords:** Mesenchymal stem cell, Exosome, miR-199a-3p, Hepatocellular carcinoma, Chemosensitivity

## Abstract

**Background:**

MiR-199a-3p (miR-199a) can enhance the chemosensitivity of hepatocellular carcinoma (HCC). Because of the easy degradation of miRNA by direct infusion, effective vehicle-mediated delivery of miR-199a may represent a new strategy for improving HCC chemotherapy. Considering mesenchymal stem cell (MSC)-derived exosomes as promising natural nanovectors for drug and molecule delivery, we aimed to determine whether exosomes from adipose tissue-derived MSCs (AMSCs) could be used to deliver miR-199a and improve HCC chemosensitivity.

**Methods:**

MiR-199a-modified AMSCs (AMSC-199a) were constructed by miR-199a lentivirus infection and puromycin selection. MiR-199-modified exosomes (AMSC-Exo-199a) were isolated from the supernatant of AMSC-199a and were assessed by transmission electron microscopy, nanoparticle tracking analysis, and flow cytometry analysis. The expression levels of miR-199a in HCC samples, AMSCs, exosomes, and HCC cells were quantified by real-time PCR. The effects of AMSC-Exo-199a on HCC chemosensitivity were determined by cell proliferation and apoptosis assays and by *i.v.* injection into orthotopic HCC mouse models with doxorubicin treatment. MTOR, p-4EBP1 and p-70S6K levels in HCC cells and tissues were quantified by Western blot.

**Results:**

AMSC-Exo-199a had the classic characteristics of exosomes and could effectively mediate miR-199a delivery to HCC cells. Additionally, AMSC-Exo-199a significantly sensitized HCC cells to doxorubicin by targeting mTOR and subsequently inhibiting the mTOR pathway. Moreover, *i.v.*-injected AMSC-Exo-199a could distribute to tumor tissue and markedly increased the effect of Dox against HCC in vivo.

**Conclusions:**

AMSC-Exo-199a can be an effective vehicle for miR-199a delivery, and they effectively sensitized HCC to chemotherapeutic agents by targeting mTOR pathway. AMSC-Exo-199a administration may provide a new strategy for improving HCC chemosensitivity.

## Background

Hepatocellular carcinoma (HCC) is the sixth most common tumor and the second most frequent cause of cancer death worldwide [1]. Aside from liver transplantation, the most common curative measure for HCC is chemotherapy. However, HCC displays high resistance to commonly used chemotherapeutic agents, such as 5-fluorouracil and doxorubicin (Dox) [2]. Therefore, the discovery of new targets and the development of novel therapeutic approaches to enhance HCC chemosensitivity are urgently needed.

MicroRNAs (miRNAs) have emerged as crucial regulatory molecules for almost every biochemical pathway in humans [3]. The progression of HCC and the acquisition of multidrug resistance are critically influenced by miRNAs through the regulation of key genes in cellular regulatory pathways [4]. Several studies have showed that a variety of miRNAs are deregulated in HCC [3, 5, 6]. MiR-199a-3p, the third most highly expressed miRNA in normal liver, is downregulated in virtually all HCCs, and its reduction correlates with poor prognosis [7, 8]. Restoration of miR-199a-3p in HCC cell lines leads to reduced cell proliferation, invasion and migration, as well as enhanced doxorubicin sensitivity by suppressing the expressions of its target genes including YAP1 [9], CD151 [10] and mTOR [11]. Thus, delivery of miR-199a-3p to HCC cells might be a potential strategy for increasing HCC chemosensitivity.

The application of nanoparticles as gene delivery systems in cancer therapy has attracted increasing attention, mainly due to their in vivo stability and biodegradability [12, 13]. Recently, increasing attention has been paid to the exosome, a membrane-bound nanosized vesicle produced by almost all cell types. Because exosomes naturally deliver nucleic acids, proteins and lipids to recipient cells, they might act as promising natural nanovectors for drugs and biological molecules [14]. Accumulating evidence has shown that exosomes have unique features as drug delivery systems, such as low immunogenicity, high biocompatibility, low toxicity, and the ability to cross the blood-brain barrier [15]. Because mesenchymal stem cells (MSCs) are efficient and prolific producers of exosomes, they can be engineered for overexpressing specific miRNAs that are incorporated into the exosomal cargo and delivered in vivo to target specific molecules in disease [16, 17].

In the present study, we investigated whether MSC-derived exosomes could act as carriers of miR-199a-3p to enhance HCC chemosensitivity in vitro and in vivo.

## Methods

### Isolation and identification of AMSCs

Subcutaneous adipose tissue was obtained from a patient undergoing tumescent liposuction at the First Affiliated Hospital in Hangzhou. This study was approved by the hospital’s ethics committee, and informed consent was obtained from the patient. Adipose tissue was processed as previously described [18], and the derived cells were maintained in a MesenCult™-ACF Plus Medium kit (STEMCELL Technologies Inc.) containing 2 mM L-glutamine (Thermo Fisher Scientific, Inc.) and 1% antibiotic-antimycotic (Thermo Fisher Scientific, Inc.). The phenotype profile of AMSCs (passages 3 to 6) was evaluated through flow cytometry analysis (BD Accuri® C6 flow cytometer) using PE-labeled cluster designation 29 (CD29), CD31, CD44, CD45, CD73, CD90, CD105, and human leukocyte antigen-DR (HLA-DR) (Biolegend) antibodies. Mouse IgG1 was used as an isotype control.

### Cell culture

The HCC cell lines, Huh7, SMMC-7721, and PLC/PRF/5 cells, and the human normal hepatocyte cell line HL-7702 were maintained in DMEM (Thermo Fisher Scientific, Inc.) containing 10% FBS (Thermo Fisher Scientific, Inc.), and 1% antibiotic-antimycotic.

### Lentivirus infection

Prior to transfection, 1 × 10^6^ AMSCs were seeded in 10 mL of MesenCultTM-ACF plus medium overnight. AMSCs were then infected with lentiviruses (MOI = 10.0) containing pre-miR-199a-3p (LV-199a) or pre-cel-miR-67 (LV-67), which contained no known mRNA-binding targets in humans (GenScript). After puromycin selection, the miRNA-modified AMSCs were harvested for real-time polymerase chain reaction (PCR) analysis.

### Isolation and identification of AMSC-derived exosomes (AMSC-Exo)

Exosomes were isolated from the AMSC supernatant by using a MagCapture™ Exosome Isolation kit (Wako) in accordance with the manufacturer’s instructions. The morphology of exosomes was observed by transmission electron microscopy (TEM), and images of the exosomes were captured using the FEI Tecnai™ Spirit (T12) TEM (Hillsboro, Oregon, USA). The particle size and exosome concentration were determined by nanoparticle tracking analysis (NTA) using NanoSight NS300 (Malvern). The exosomes were then characterized by flow cytometry analysis of exosome surface markers by using Exosome-Human CD9, CD63 and CD81 Isolation/Detection kits (Thermo Fisher Scientific, Inc.) according to the manufacturer’s instructions. Mouse IgG1 was used as an isotype control. The protein content of exosomes was determined by using a BCA protein assay kit (Pierce). Subsequently, exosome pellets were resuspended in sterile PBS at a total protein concentration of 5 μg/μL.

### Isolation and detection of miRNA

Total RNA enriched with miRNAs was isolated from AMSC-Exo, AMSC-Exo-treated cells, and tissue samples of AMSC-Exo-treated mice by using a miRVana miRNA isolation kit (Thermo Fisher Scientific, Inc.) according to the manufacturer’s instructions. Complementary DNA was synthesized from the isolated miRNAs by using TaqMan™ hsa-miR-199a-3p-specific primers (Thermo Fisher Scientific, Inc.) and a TaqMan™ MiRNA Reverse Transcription kit (Thermo Fisher Scientific, Inc.). Real-time PCR was then performed according to the manufacturer’s instructions (Thermo Fisher Scientific, Inc.) to examine miR-199a-3p expression. Data were normalized to the average cycle threshold (CT) value of U6, and the 2^-ΔΔCT^ method was used to determine relative miRNA expression.

### Confocal microscopy detection

AMSCs were labeled with the phospholipid membrane dye, lipophilic carbocyanine DilC_16_(3) (1.25 μM). After 10 min of incubation at 37 °C, cells were washed and were resuspended in fresh media for 48 h. Fluorescent exosomes were collected and were added to recipient PLC/PRF/5 cells. Afterward, cells were fixed with methanol, mounted on slides, and imaged via confocal microscopy (Olympus). Background fluorescence was subtracted using unstained cells.

### Western blot analysis

After treatment with AMSC-Exo or transfection with the mTOR expression plasmid, HCC cells or tumor samples were lysed with RIPA peptide lysis buffer (Beyotime Biotechnology, Jiangsu, China) containing 1% protease inhibitors (Pierce). The protein content of different fractions was determined via the BCA method. Equivalent amounts of protein (20 μg) were separated by SDS-PAGE with 10% gels and then were transferred to polyvinylidene difluoride membranes (Millipore, Bedford, MA) and blocked with 1% BSA in TBST for 1 h at room temperature. The membranes were incubated with mTOR and phosphorylated-4EBP1 and -70S6K or GAPDH (Abcam) antibodies overnight at 4 °C. After washing, the membrane was incubated with an HRP-conjugated secondary antibody (1:3000; Abcam) for 1 h. Protein bands were identified using an enhanced chemiluminescence system and were visualized using a the ChemiScope Western Blot Imaging System (Clinx Science Instruments Co., Ltd). The gray value assay was performed by using ImageJ software (Rawak Software, Inc. Germany).

### Real time cellular analysis (RTCA)

An xCELLigence DP device (Roche Diagnostics, Germany) was used to monitor cell proliferation in real time. HCC cells (5 × 10^5^) were seeded in 96-well electronic microtiter plates (Roche Diagnostics, Germany) and then were treated with Dox combined with AMSC-Exo-199a or AMSC-Exo-67. The xCELLigence system was used to measure all cells for 96 h according to the instructions. A programmed signal detector was used to measure the cell density in quadruplicate every 30 min. RTCA software (version 1.2) from Roche Diagnostics was used for collecting and analyzing data. For the 50% inhibitory concentration (IC50) assay, data were analyzed by GraphPad Prism 5 software (GraphPad, La Jolla, CA).

### Cell apoptosis analysis

Cells were plated in 6-well plates at a concentration of 2 × 10^5^/well and were treated with 10 μM Dox with or without AMSC-Exo (50 ng/μL). Cell apoptosis was analyzed by using an Annexin V assay kit (BD, USA) and then measured by a Beamcyte-1026® flow cytometer (BDA Inc., China).

### Orthotopic HCC mouse model and exosome treatment

Male BALB/c nude mice (6 weeks old) were purchased from Zhejiang Academy of Medical Science and were reared under a specific pathogen-free condition. All experimental procedures were reviewed and approved by the Institutional Animal Care and Use Committee of the First Affiliated Hospital of Zhejiang University. PLC/PRF/5 cells (1 × 10^7^) were subcutaneously inoculated into the right flanks of two male BALB/c nude mice. After one month, the subcutaneous ectopic tumors about 1 cm in diameter were harvested and then cutted into 1–2 mm^3^ sections under aseptic conditions. Following anesthesia, the tumor sections were replanted into the liver lobe of new nude mice using ophthalmic ligature forceps to construct a liver orthotopic HCC mouse model. Two weeks after implantation, mice were randomized into groups of 6 mice prior to exosome treatment. MiRNA-modified AMSC-Exo (50 μg total protein in 200 μl PBS) was administered into these mice via tail vein injection combined with or without Dox treatment (10 mg/kg) once per week. In vivo bioluminescent imaging to determine tumor burden was performed with a Lumina imaging system (Nippon Roper, I.C.E., Tokyo, Japan). Ten minutes before imaging, mice were injected intraperitoneally with 150 mg/kg luciferin. Images were collected and analyzed by Living Image 4.1 (PerkinElmer, Waltham, MA, USA) and SlideBook 5 (Intelligent Imaging Innovations, Denver, CO, USA) software. At the experimental end point, the liver samples were collected and evaluated by hematoxylin-eosin staining (HE) and Western blot analysis. For fluorescent detection by confocal microscopy, AMSC-Exo was stained with PKH26 before administration.

### Statistical analysis

Differences between groups were analyzed by using conventional Student’s t-test or ANOVA. Each experiment was repeated at least three times, and the data were presented as the mean ± SD (standard deviation). The results were considered significant at *P < 0.05, **P < 0.01.

## Results

### MiR-199a-3p level is related to chemosensitivity of HCC cells

**Fig. 1:**
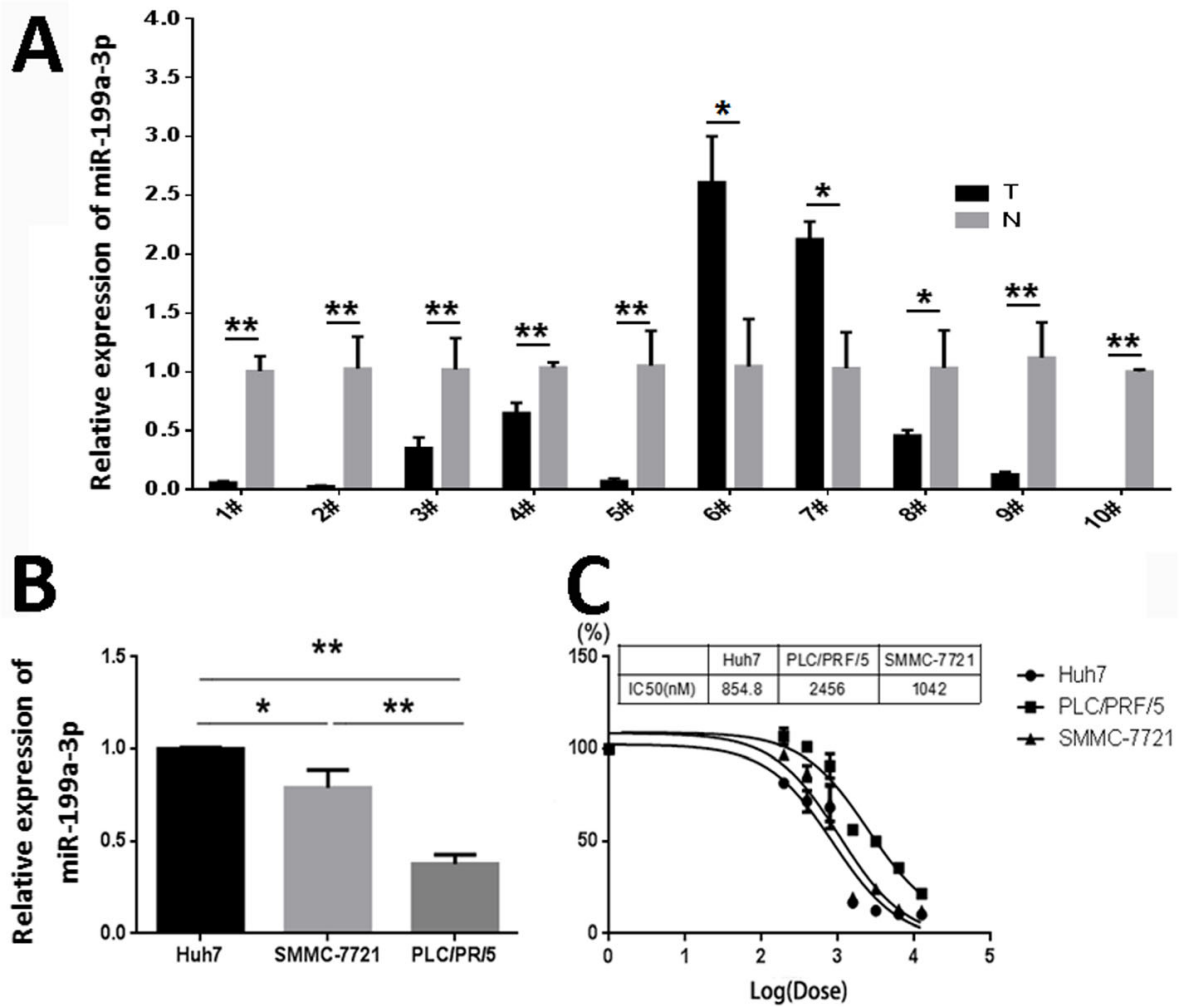
MiR-199a-3p expression is inversely correlated with HCC chemosensitivity. **a** The expression levels of miR-199a-3p were detected by real time-PCR in HCC tissues (T) and the paired adjacent non-cancerous liver tissues (N). **b** Real time-PCR detection of miR-199a-3p expression in HCC cell lines. **c** At 96 h after treated with Dox, the IC50 value of HCC cells against Dox were analyzed by GraphPad Prism 5 software based on the data from real time cellular analysis. Data are presented as mean ± S.D. (*P < 0.05, **P < 0.01, *n* = 3)

By examining the miR-199a-3p expression in 10 randomly selected HCC tissues and paired adjacent noncancerous liver tissues, we found that 8 out of 10 HCCs (80%) had decreased miR-199a-3p expression compared with that of the corresponding noncancerous hepatic tissues (Fig. 1a). Further assays showed that the expression level of miR-199a-3p in HCC cell lines (Fig. 1b), Huh7, SMMC-7721 and PLC/PRF/5, was correlated with the chemosensitivity of HCC cells. The IC50 value of Dox for HCC cells was highest in PLC/PRF/5 cells (Fig. 1c), which had the lowest expression level of miR-199a-3p.

### AMSC-Exo mediates miR-199a-3p transfer into HCC cells

**Fig. 2:**
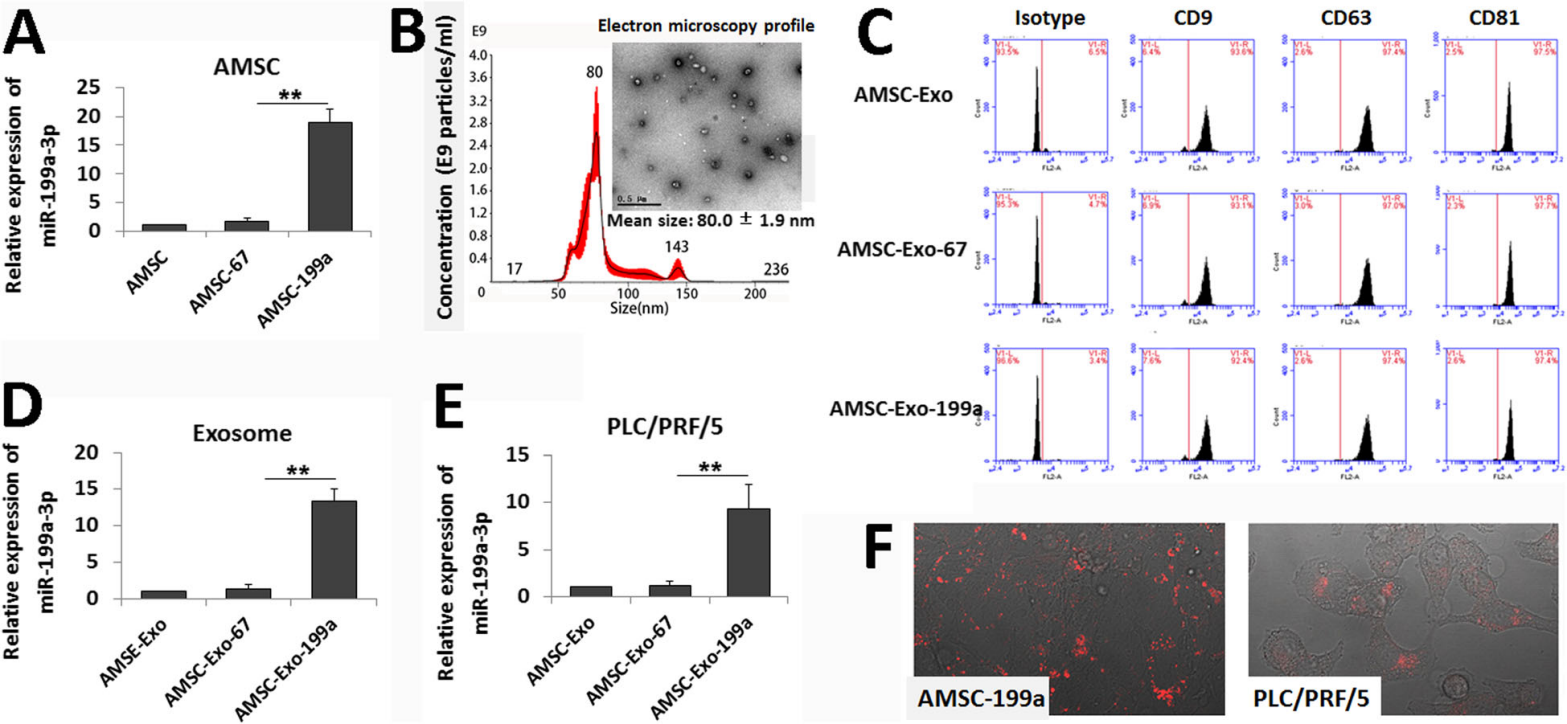
AMSC-Exo-mediated miR-199a-3p transfer into HCC cells. **a** Real-time PCR detection of miR-199a-3p expression in navie AMSCs and miRNA-modified AMSCs. **b** Transmission electron microscopy detection on AMSC-Exo morphology and nanoparticle tracking analysis on exosome particle size and concentration (Scale bar = 0.5 μm). **c** Flow cytometric analysis of the surface markers of the exosomes derived from navie AMSCs and miRNA-modified AMSCs. **d** Real-time PCR detection of miR-199a-3p expression in AMSC-Exo. **e** Real time-PCR detection of miR-199a-3p expression in AMSC-Exo-treated PLC/PRF/5 cells. **f** Confocal images of AMSC-199a stained with DilC_16_(3) and the PLC/PRF/5 cells incubated by the exosomes derived from DilC_16_(3)-stained AMSC-199a. Data are presented as mean ± S.D. (**P < 0.01, *n* = 3)

To determine whether AMSC-derived exosomes could be used as effective vehicles for miR-199a-3p delivery to improve HCC chemotherapy, AMSCs were modified with miR-199a-3p (AMSC-199a) by LV-199a infection, and elevated miR-199a-3p level in AMSC-199a was confirmed by qRT-PCR (Fig. 2a). Exosomes were then isolated from the culture supernatant of AMSC-199a. TEM showed the classic exosomal morphology of AMSC-199a-derived exosomes (AMSC-Exo-199a), and NTA of AMSC-Exo-199a showed a size distribution with a mean diameter of 80.0 ± 1.9 nm (Fig. 2b). Flow cytomerty assays also confirmed that AMSC-Exo-199a expressed exosomal markers, such as CD9, CD63, and CD81 (Fig. 2c). In addition, a real-time PCR assay showed that the expression of miR-199a-3p in AMSC-Exo-199a was 10.9 ± 1.6-fold higher than that in AMSC-Exo-67, which was derived from cel-miR-67-modified AMSCs (AMSC-67) (Fig. 2d).

Furthermore, to assess the role of AMSC-Exo on miR-199a-3p communication, we analyzed the miR-199a-3p level in PLC/PRF/5 cells after incubation with AMSC-Exo-199a for 24 h. As we expected, the miR-199a-3p expression was dramatically elevated (9.8 ± 1.1-fold) in the PLC/PRF/5 cells following AMSC-Exo-199a treatment, whereas few changes were observed following AMSC-Exo-67 treatment (Fig. 2e). Additionally, by incubation with DilC_16_(3)-labeled AMSC-Exo, the fluorescent membrane dye enabled detection in unlabeled recipient HCC cells (Fig. 2f). These data indicate that AMSC-Exo-199a can be effective vectors for miR-199a-3p transfer.

### AMSC-Exo-199a increases chemosensitivity of HCC cells

**Fig. 3:**
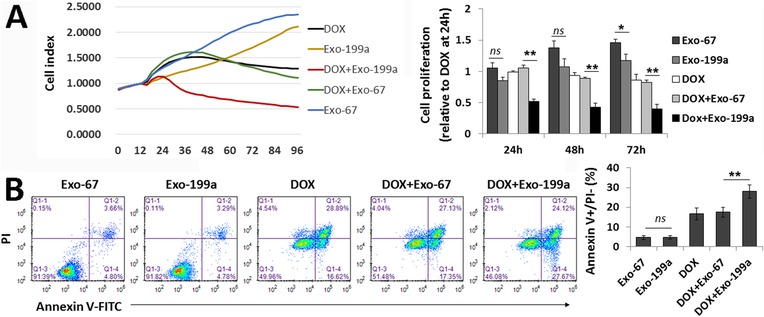
AMSC-Exo-199a sensitizes PLC/PRF/5 cells to doxorubicin. **a** The cell vialibility of PLC/PRF/5 cells by doxorubicin (Dox) expusure were determined by real time cellular analysis. The relative cell proliferation ratio was normalized over the cell index of the PLC/PRF/5 cells with Dox-treatment alone at 24 h. **b** FITC-Annexin V/PI stain for cell apoptosis assay on AMSC-Exo-treated PLC/PRF/5 cells combined with or without Dox expusure. AMSC-Exo-199a could significantly increase the percent of apoptotic cells (Annexin V+/PI-) by Dox expusure. Data are presented as mean ± S.D. (*P < 0.05, **P < 0.01, *ns* = nonsense, n = 3). *Exo-199a* AMSC-Exo-199a, *Exo-67* AMSC-Exo-67

To determine whether AMSC-Exo-199a could affect the chemosensitivity of HCC cells, RTCA and flow cytometry were performed to assess cell viability and apoptosis, respectively. As shown in Fig. 3a, the viability of Dox-treated PLC/PRF/5 cells was remarkably decreased when cells were treated in combination with AMSC-Exo-199a compared to that of cells treated with in combination with AMSC-Exo-67, while treatment with AMSC-Exo-199a alone (without Dox exposure) just slightly reduced PLC/PRF/5 cell proliferation compared with AMSC-Exo-67 treatment alone at 72 h. Annexin V/PI staining also showed a significant increase in the portion of apoptotic cells following Dox exposure in AMSC-Exo-199a-treated PLC/PRF/5 cells compared to that of AMSC-Exo-67-treated cells, whereas treatment with AMSC-Exo-199a alone had no obvious effect on apoptosis (Fig. 3b). These data indicate that AMSC-Exo-199a can be used to improve the chemosensitivity of HCC cells.

### AMSC-Exo-199a affects mTOR pathway in HCC cells

**Fig. 4:**
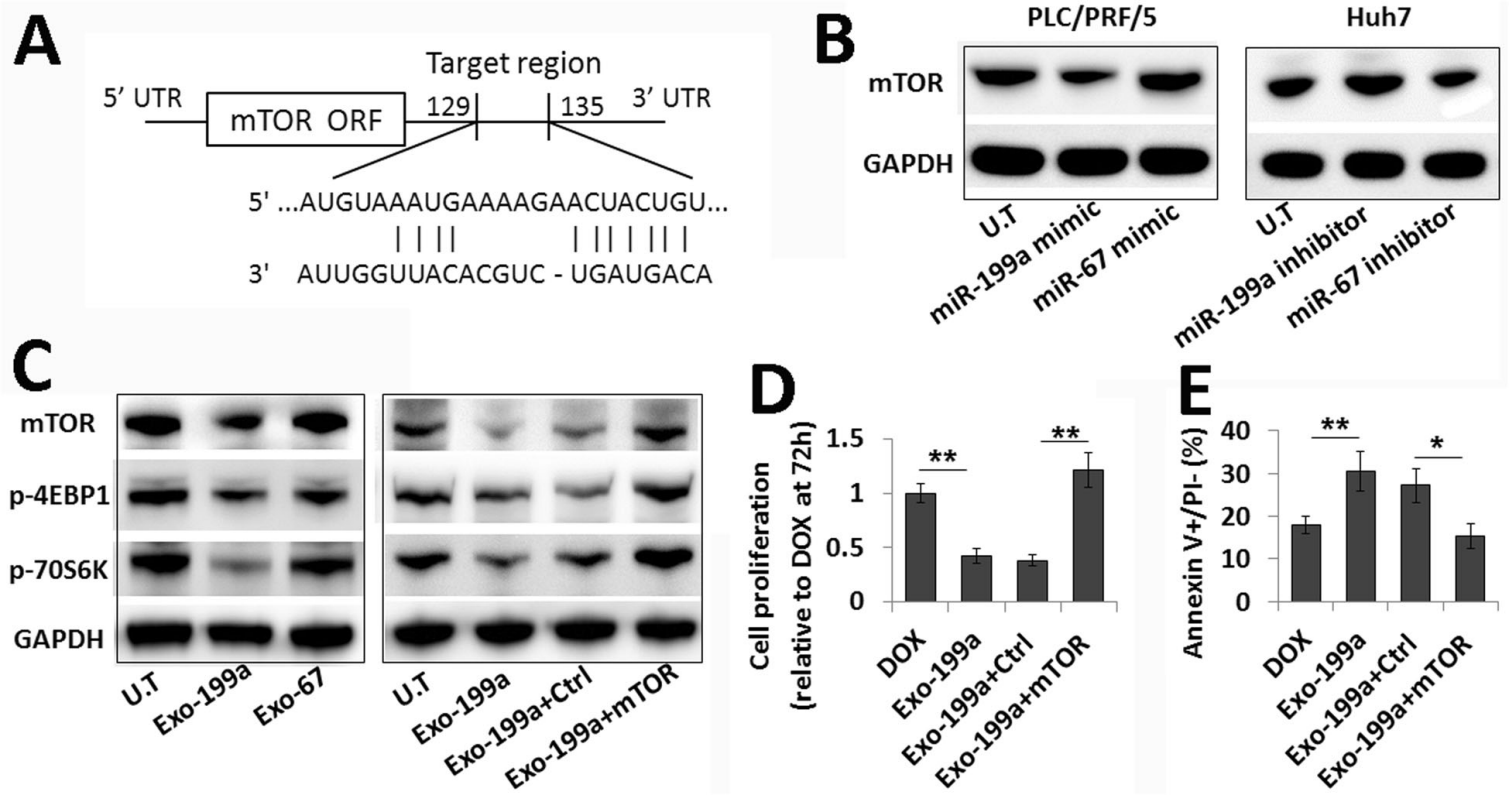
mTOR pathway involved in AMSC-Exo-199a-enhanced HCC chemosensitivity. **a** The target site of miR-199a-3p in 3′-UTR of mTOR mRNA. **b** Western blot analysis of mTOR expression in PLC/PRF/5 and Huh7 cells transfected with miR-199a-3p mimics or inhibitors, respectively. MiR-67 mimics or inhibitors transfection were used as controls. **c** Western blot analysis of the expression level of mTOR and the phosphorylation level of 4EBP1 and 70S6K in PLC/PRF/5 cells with AMSC-Exo treatment and those in mTOR plasmid-transfected PLC/PRF/5 cells with AMSC-Exo treatment. **d** The relative cell proliferation ratio were calculated by normalizing over the cell index of the PLC/PRF/5 cells with Dox-treatment alone at 72 h. **e** FITC-Annexin V/PI stain revealed a reversion of the AMSC-Exo-199a-improved chemosensitivity of PLC/PRF/5 cells by mTOR transfection. Data are presented as mean ± S.D. (*P < 0.05, **P < 0.01, n = 3). *Exo-199a* AMSC-Exo-199a, *Exo-67* AMSC-Exo-67

MTOR, as an oncogene, is known to be related to tumor chemo-resistance [19]. Recently, mTOR was identified as a direct target of miR-199a-3p (Fig. 4a). Transfection with miR-199a mimics reduced mTOR expression in HCC cells, while transfection with miR-199a inhibitors increased mTOR expression in HCC cells (Fig. 4b). To determine whether AMSC-Exo-199a-induced chemosensitivity via targeting mTOR, we analyzed the mTOR expression level in HCC cells following AMSC-Exo treatment. Compared to AMSC-Exo-67-treated HCC cells, the mTOR expression was consistent with the phosphorylation level of its downstream proteins, 4EBP1 and 70S6K, which were all significantly decreased in AMSC-Exo-199a-treated cells (Fig. 4c). Moreover, the AMSC-Exo-199a-reduced phosphorylation of 4EBP1 and 70S6K could be reversed by transfection with an mTOR overexpression plasmid (Fig. 4c). Restoration of mTOR expression could also reverse the effect of AMSC-Exo-199a in promoting chemosensitivity of HCC cells, which was determined by cell proliferation and apoptosis assays (Fig. 4d and e). The involvement of mTOR in AMSC-Exo-199a-enhanced HCC chemosensitivity was also shown by experiments with rapamycin, an mTOR inhibitor. As expected, rapamycin significantly inhibited mTOR activation and the subsequent phosphorylation of 4EBP1 and 70S6K in HCC cells. It also markedly increased the sensitivity of HCC cells to Dox, which was similar to the effect of AMSC-Exo treatment (Additional file 1: Figure S1).

These data further suggest that AMSC-Exo-199a enhances the chemosensitivity of HCC cells by targeting mTOR pathway.

### AMSC-Exo-199a sensitizes HCC to Dox in vivo

**Fig. 5:**
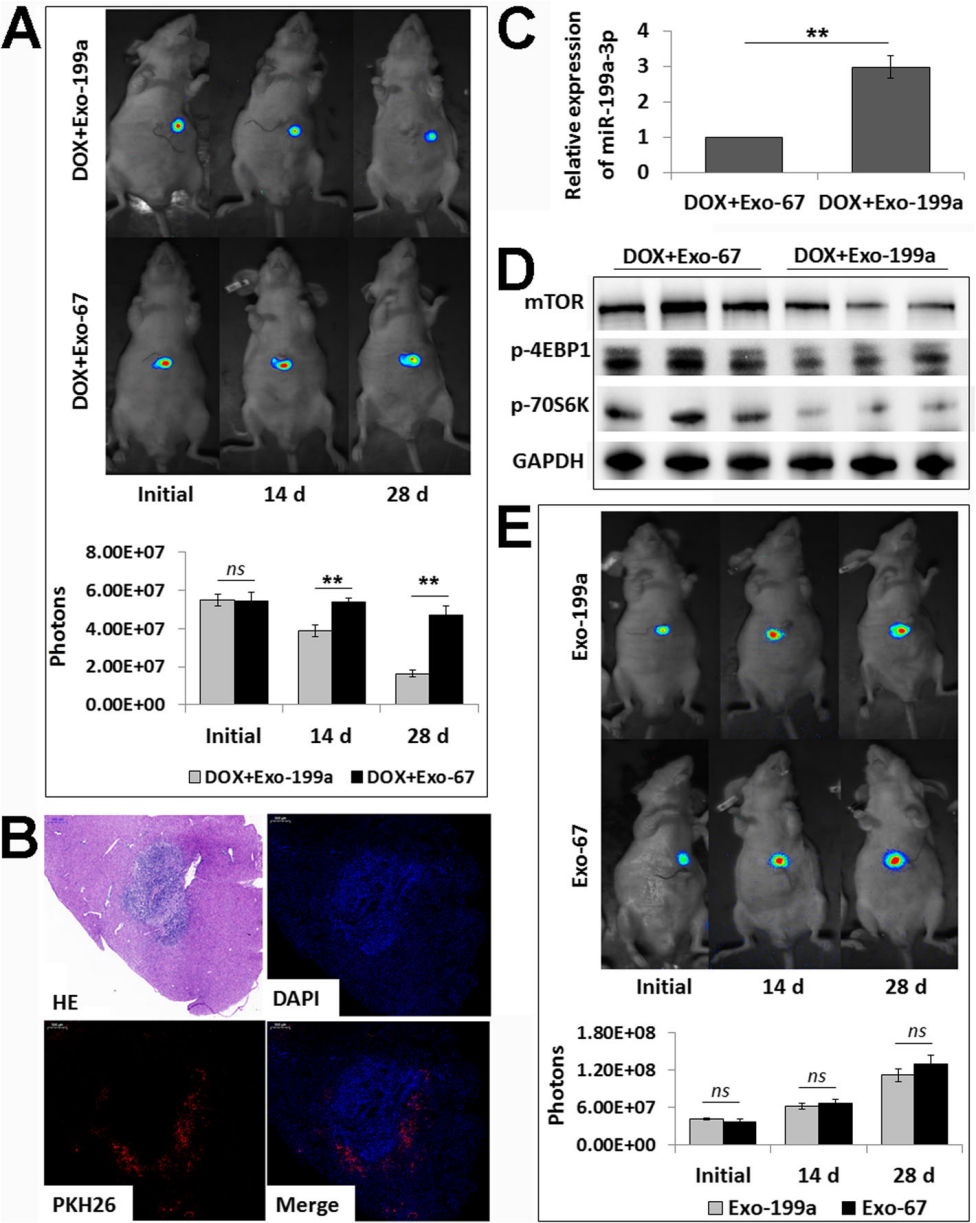
AMSC-Exo-199a sensitizes HCC to Dox in vivo by suppressing mTOR pathway. **a** Tumor growth in Dox-treated mice was measured by whole-mount imaging of luciferin florescence and photon counts were analyzed at initial and 14 d and 28 d after AMSC-Exo-199 or AMSC-Exo-67 *i.v.* injection. **b** Hematoxylin-eosin staining (HE) and fluorescent detection of liver samples collected at 6 h after AMSC-Exo *i.v.* injection. **c** Real-time PCR detection of miR-199a-3p expression in the liver samples of mice with AMSC-Exo *i.v.* injection. **d** Western blot analysis of mTOR, p-4EBP1 and p-70S6K expression levels in HCC samples of the mice with AMSC-Exo and Dox combined therapy. **e** Tumor growth in AMSC-Exo monotherapy mice was measured as above at initial and 14 d and 28 d after AMSC-Exo-199 or AMSC-Exo-67 *i.v.* injection. Data are presented as mean ± S.D. (**P < 0.01, *ns* = nonsense, *n* = 6, 3 from 6 samples were shown in **d**). *Exo-199a* AMSC-Exo-199a, *Exo-67* AMSC-Exo-67

Finally, to further determine whether AMSC-Exo-199a could sensitize HCC cells to chemotherapeutic agents in vivo, AMSC-Exo-199a (50 μg of total protein in 200 μl of PBS) was administered to an orthotopic HCC mouse model via tail vein injection combined with Dox treatment. Tumor growth was monitored through measuring luciferase emission every two weeks. As shown in Fig. 5a, the combined therapy of AMSC-Exo-199a and Dox, compared to the combination of AMSC-Exo-67 and Dox, produced significant tumor growth retardation in treated mice. In addition, we found that the *i.v.* injected AMSC-Exo-199a labeled with PKH26 mainly distributed around the HCC (Fig. 5b), and it significantly increased the miR-199a-3p level in tissue samples (Fig. 5c). Moreover, the expression levels of mTOR and phosphorylated 4EBP1 and 70S6K were remarkably reduced in the HCC samples of mice with combined treatment of AMSC-Exo-199a and Dox compared with that of the mice treated with AMSC-Exo-67 and Dox (Fig. 5d).

However, no statistically significant difference was observed between the AMSC-Exo-199a and AMSC-Exo-67 monotherapy groups (without combination with Dox) on HCC growth inhibition at 28 days after AMSC-Exo administration (Fig. 5e).

Overall, these results revealed that AMSC-Exo-199a administration promotes the growth inhibitory effects of Dox on HCC cells.

## Discussion

Due to their role in cell-to-cell communication and loading exogenous cargo, exosomes are considered natural nanocarriers that can be used in clinical applications, such as drug delivery or transfer of specific mRNAs, regulatory miRNAs, lipids, and proteins [15, 20]. Exosomes are produced by a variety of cell types. Of the cell types known to produce exosomes, the readily available proliferative, immunosuppressive and clinically tested human MSCs are the most prolific producer [16]. Exosome-mediated transfer of miR-133b from MSCs to neural cells stimulated neurite growth [21]. MSC-derived exosomes that expressed miR-146b inhibited glioma growth [22]. The present study provided a novel strategy for increasing HCC chemosensitivity through AMSC-Exo-mediated transfer of therapeutic miR-199a-3p. The miR-199a-3p-modified AMSC-Exo can effectively mediate miR-199a-3p transmission between AMSCs and HCC cells and then increase the sensitivity of HCC cells to chemotherapeutic agents by targeting mTOR pathway.

The mTOR pathway is a central regulator of mammalian metabolism and physiology [23]. Overactivation of mTOR signaling significantly contributes to the initiation and development of tumors and mTOR activity was found to be deregulated in many types of cancer including HCC [24, 25]. MTOR interacts with other proteins and serves as a core component of two distinct protein complexes, mTOR complex 1 (mTORC1) and mTOR complex 2, which regulate different cellular processes. Notable downstream targets of mTORC1 are ribosomal protein S6 kinase (70S6K) and eukaryotic translation initiation factor 4E binding proteins (4EBPs) [23, 26]. Recent studies have proposed an important role for mTOR activation in chemo-resistance [19, 27]. Thus, mTOR signaling appears to be a major compensatory pathway conferring resistance to targeted therapies. For example, Zhang et al. showed that mTOR inhibition by INK128 abolished chemoresistance to doxorubicin in neuroblastoma cells [28]. Peng et al. showed that AZD-2014, a novel mTOR kinase inhibitor, dramatically sensitized human HCC cells to resminostat, leading to profound levels of cytotoxicity and apoptosis [29]. In the present study, we showed that AMSC-Exo-199a could effectively sensitize HCC to Dox by suppressing mTOR signaling, as determined by decreased expression levels of mTOR and phosphorylated 4EBP1 and 70S6K in HCC cells. That miR-199a-3p-mediated regulation of mTOR can influence HCC chemosensitivity was also shown in a previous study. Our study further provided a method for delivering miR-199a-3p by AMSC-Exo-mediated association with HCC cells, which then regulated mTOR signaling.

In addition to mTOR, hexokinase 2 (HK2), pyruvate kinase M2 (PKM2), p21 activated kinase 4 (PAK4), yamaguchi sarcoma viral homolog 1 (YES1), integrin β8 (ITGB8), and mitochondrial transcription factor A (TFAM) were all reported to be direct targets of miR-199a-3p. MiR-199a-3p-mediated regulation of these genes has been shown to correlate with various types of cancer, including HCC [30–34]. Thus, we propose that AMSC-Exo-199a might also promote HCC chemosensitivity by regulating these above genes. We further examined the expression levels of HK2, PKM2, PAK4, YES1, ITGB8 and TFAM in HCC cell lines. However, there were no obvious correlations between the expression of these with miR-199a-3p levels in HCC cells and the chemosensitivity of HCC cells (data not shown). These results suggest that mTOR might be the main target of miR-199a-3p in HCC cells and that it might play a key role in miR-199a-3p-associated HCC chemosensitivity. Moreover, mTOR overexpression could reverse both of the AMSC-Exo-199a-reduced phosphorylation of 4EBP1 and 70S6K in HCC cells and the reduced chemoresistance of HCC cells by AMSC-Exo-199a treatment, further confirming that AMSC-Exo-199a enhances HCC chemosensitivity does by suppressing mTOR expression and its subsequent signaling activation.

Our previous study has shown that intratumor injection of miR-122-modified AMSC-Exo (AMSC-Exo-122) could significantly increase the antitumor efficacy of chemotherapeutic agents on HCC in a subcutaneous tumor bearing model [18]. In the present study, we further showed that the *i.v.* injection of AMSC-Exo-199a led to it mainly being distributed at the tumor tissues and effectively enhanced the chemosensitivity of HCC in an orthotopic model. The ability of AMSC-Exo-199a to address the tumor microenvironment may improve the feasibility of AMSC-Exo-based therapy in clinical applications. However, treatment with AMSC-Exo-199a alone (without combination with Dox) could not effectively suppress HCC growth. This may due to the limited dose of AMSC-Exo-mediated miR-199a-3p transfer just by *i.v.* injection was insufficient for HCC growth inhibition. Further optimization of the strategies of AMSC-Exo modification and infusion route, timing and dose may further improve the efficacy of AMSC-Exo-199a against HCC.

## Conclusions

This study showed that miR-199a-3p-modified AMSC-Exo can effectively increase the sensitivity of HCC cells to chemotherapeutic agents by targeting mTOR pathway. AMSC-Exo-199a administration may provide a new strategy for improving HCC chemosensitivity.

## Supplementary information

**Additional file 1: Figure S1.** Involvement of mTOR pathway in AMSC-Exo-199a-enhanced HCC chemosensitivity.

## Data Availability

All data generated or analyzed during this study are included either in this article or in the additional files.

## Abbreviations

4EBP1: 4E binding protein 1
70S6K: Ribosomal protein S6 kinase
AMSC: Adipose tissue-derived MSC
AMSC-199a: miR-199a-modified AMSCs
AMSC-67: Cel-miR-67-modified AMSCs
AMSC-Exo: AMSC-derived exosomes
AMSC-Exo-199a: AMSC-199a-derived exosomes
AMSC-Exo-67: AMSC-67-derived exosomes
CD151: Cluster designation 151
CT: Cycle threshold
Dox: Doxorubicin
HCC: Hepatocellular carcinoma
HE: Hematoxylin-eosin
HK2: Hexokinase 2
IC50: 50% inhibitory concentration
ITGB8: Integrin β8
LV: Lentivirus
miRNA: MicroRNA
MSC: Mesenchymal stem cell
NTA: Nanoparticle tracking analysis
PAK4: p21 activated kinase 4
PCR: Polymerase Chain Reaction
PKM2: Pyruvate kinase M2
RTCA: Real Time Cellular Analysis
TEM: Transmission electron microscopy
TFAM: Mitochondrial transcription factor A
YAP1: Yes associated protein 1
YES1: Yamaguchi sarcoma viral homolog 1
