# Supplementary material for: MiR-199a-modified exosomes from adipose tissue-derived mesenchymal stem cells improve hepatocellular carcinoma chemosensitivity through mTOR pathway
Source: J Exp Clin Cancer Res. 2020 Jan 2;39:4. doi: 10.1186/s13046-019-1512-5 (PMC6941283; doi:10.1186/s13046-019-1512-5)
Supplement: Supplementary file 1 — Additional file 1: Figure S1. Involvement of mTOR pathway in AMSC-Exo-199a-enhanced HCC chemosensitivity. [file 13046_2019_1512_MOESM1_ESM.docx]

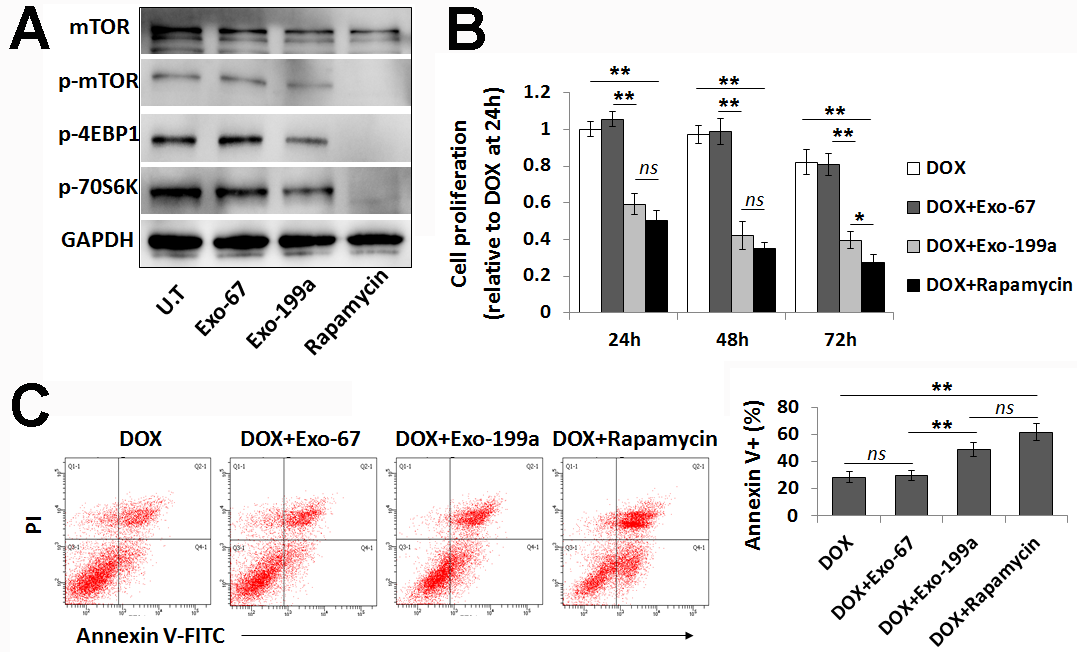


**Figure S1** Involvement of mTOR pathway in AMSC-Exo-199a-enhanced HCC chemosensitivity

**a** Western blot analysis of the expression level of mTOR and the phosphorylation level of 4EBP1 and 70S6K in PLC/PRF/5 cells with AMSC-Exo or rapamycin treatment. **b** The cell vialibility of PLC/PRF/5 cells by doxorubicin (Dox) expusure were determined by real time cellular analysis. The relative cell proliferation ratio was normalized over the cell index of the PLC/PRF/5 cells with Dox-treatment alone at 24 h. **c** FITC-Annexin V/PI stain for cell apoptosis assay on Dox-treated PLC/PRF/5 cells combined with AMSC-Exo or rapamycin. (**P* < 0.05, ***P* < 0.01, *ns*=nonsense, n = 3). *Exo-199a* AMSC-Exo-199a, *Exo-67* AMSC-Exo-67.
